# Ionisation processes and laser induced periodic surface structures in dielectrics with mid-infrared femtosecond laser pulses

**DOI:** 10.1038/s41598-020-65613-w

**Published:** 2020-05-26

**Authors:** George D. Tsibidis, Emmanuel Stratakis

**Affiliations:** 1 Institute of Electronic Structure and Laser (IESL), Foundation for Research and Technology (FORTH), N. Plastira 100, Vassilika Vouton, 70013 Heraklion, Crete Greece; 2 Department of Physics, University of Crete, 71003 Heraklion, Greece

**Keywords:** Condensed-matter physics, Computational methods

## Abstract

Irradiation of solids with ultrashort pulses and laser processing in the mid-Infrared (mid-IR) spectral region is a yet predominantly unexplored field with a large potential for a wide range of applications. In this work, laser driven physical phenomena associated with processes following irradiation of fused silica (SiO_2_) with ultrashort laser pulses in the mid-IR region are investigated in detail. A multiscale modelling approach is performed that correlates conditions for formation of perpendicular or parallel to the laser polarisation low spatial frequency periodic surface structures for low and high intensity mid-IR pulses (not previously explored in dielectrics at those wavelengths), respectively. Results demonstrate a remarkable domination of tunneling effects in the photoionisation rate and a strong influence of impact ionisation for long laser wavelengths. The methodology presented in this work is aimed to shed light on the fundamental mechanisms in a previously unexplored spectral area and allow a systematic novel surface engineering with strong mid-IR fields for advanced industrial laser applications.

## Introduction

The employment of ultra-short pulsed laser sources for material processing has received considerable attention over the past decades due to the important technological applications, particularly in industry and medicine^1–7^. There is a variety of surface structures generated by laser pulses and more specifically, the so-called laser-induced periodic surface structures (LIPSS) on solids that have been studied extensively^8–19^ and are related to those applications. A range of LIPSS types have been produced based on the material and the laser parameters^6,9,16,20,21^. According to the morphological features of the induced surface structures such as their periodicity and orientation, LIPSS can be classified in: (a) High Spatial Frequency LIPSS (HSFL)^16,22^, (b) Low Spatial Frequency LIPSS (LSFL)^10,23^, (c) Grooves^18,21^, (d) Spikes^18^ and (e) complex ones^20,21,24^. The LIPSS fabrication technique as well as the associated laser driven physical phenomena have been the topic of an extensive investigation. This is due to the fact that the technique constitutes a precise, single-step and scalable method to fabricate highly ordered, multi-directional and complex surface structures that mimic the unique morphological features of certain species found in nature, an approach which is usually coined as biomimetics. A thorough knowledge of the fundamental mechanisms that lead to the LIPSS formation provides the possibility of generating numerous and unique surface biomimetic structures^2,6,20,25–27^ for a range of applications, including microfluidics^1,28^, tribology^29–31^, tissue engineering^5,28^ and advanced optics^6,32^.

On the other hand, a key characteristic of the investigation of the underlying physical processes that account for LIPSS formation is that it has been centred on laser pulses in a spectral region between the visible and near-infrared frequencies (*λ*_*L*_ < 1.5 μm)^21,33^. Nevertheless, there is recently an increasing interest in the nano/micro sized patterning of materials with longer wavelengths. The motivation to explore the response of the irradiated materials and relevant surface effects in the mid-IR region originates from the challenging opportunities in photonics for mid-IR radiation^34–36^. Mid-IR has proven to constitute a spectral region that provides numerous possibilities for both fundamental and applied research^37^. For example, irradiation with intense mid-IR leads to novel phenomena like photon acceleration in metasurfaces to generation of attosecond pulses, and demonstration of megafilamentation in atmosphere, which may open doors to many exciting new applications^38^. Similarly, mid-IR-assisted damage can be used for the sub-surface micromachining of multi-layer materials, such as the inscription of silicon waveguides operating in the telecommunications band^39^. On the other hand, an abundance of molecules have impressive absorption features in the mid-IR region which makes them highly useful for applications in biomolecular sensing, explosives detection, biomedical applications, and environmental sensing^40^.

Despite the above exciting possibilities and the advances in the investigation of behaviour of irradiated material with mid-IR pulses^37–39,41–44^, there are still many crucial questions that have yet to be addressed. A fundamental question is, however, whether the underlying physics that characterises laser-matter interaction for mid-IR differs from that at lower spectral regions^45–48^. In a previous report, results showed that silicon is transparent in this region while it absorbs strongly during the pulse duration^43^. Furthermore, a systematic analysis of the role of excitation levels for conditions directly related to formation of sub-wavelength LIPSS through the investigation of surface plasmon excitation-based mechanisms revealed significantly different effects, such as surface plasmons (SP) with smaller confinement, longer lifetime and larger decay lengths for mid-IR pulses compared to irradiation with lower wavelengths^43^. These results demonstrate the need to describe in more detail the ultrafast dynamics following excitation with mid-IR sources as they can potentially influence the morphological changes on the material^43^. Another important characteristic of irradiation of solids with mid-IR sources is that as electron cycle average energy scales as ${\lambda }_{L}^{2}$ (*λ*_*L*_ stands for the laser wavelength), excited electrons are expected to gain enough energy to modify damage mechanisms at long wavelengths. Therefore, a detailed description of the physical processes that characterise interaction of laser sources with solids is crucial for efficient laser-based machining in the mid-IR region (*λ*_*L*_ > 2 μm).

One type of material which is of paramount importance for the design of optics in high power laser systems, is fused silica. While an extensive research has been conducted towards elucidating laser-induced damage in fused silica through the investigation of processes related to irradiating SiO_2_ with IR pulses^6,11,12,17,22,48–54^, little is known about the effects of electron excitation with pulses of longer wavelengths^55^. To account for the influence of mid-IR photons on the physical processes related to excitation conditions as well as possible surface modification-related mechanisms, a number of critical factors should be considered: (i) the energy absorption and excitation at larger *λ*_*L*_, (ii) the significance of nonlinear processes such as Kerr effect, multi-photon absorption, tunneling effect and impact ionisation, (iii) the role of the wavelength value in the modulation of the optical parameters, and (iv) the conditions that lead to surface patterning. In regard to the surface modification, LSFL structures with orientation parallel to that of the electric field of the incident beam (termed here as LSFLǁ) have been predicted and observed for moderate intensities at lower wavelengths^11,22,48,53^; these predictions were based on Sipe’s theory^19^ which proposes that surface patterning results from inhomogenous energy absorption from the solid due to surface roughness. Similarly, LSFL structures with orientation perpendicular to that of the electric field of the incident beam (termed here as LSFL ⊥) have also been observed in dielectrics, when intense laser beams can potentially lead to excitation levels for which a transition of the dielectric material to a ‘metallic’ state can be achieved^22,53,56^. Theoretical simulations which are validated with experimental results support the proposed scenario that the interference of the incident light with the far-field of rough non-metallic or metallic surfaces accounts for the formation of both LSFLǁ and LSFL ⊥^22,53^. On the other hand, for metallic surfaces, SP-related processes have been, also, proposed to successfully describe LSFL ⊥ structures with periodicities ~*λ*_*L*_^10,57^. Therefore, an interesting question that needs to be explored is whether conditions for the formation LSFLǁ and LSFL ⊥ can, also, be achieved following irradiation of SiO_2_ with mid-IR sources.

In this paper, we report on the excitation of SiO_2_ following irradiation with ultrashort (femtosecond) pulses at various wavelengths in the mid-IR spectral region (between *λ*_*L*_ = 2 μm and *λ*_*L*_ = 4 μm). The photoionisation rates are computed and the role of tunneling effect is highlighted. The impact of Kerr effect and nonlinearities in the refractive index *n* of the material as well as the resulting electron densities are analysed. The ultrafast dynamics of the excited carriers is described in the presence of metastable self-trapped exciton (STE) states^49^. Formation of LSFLǁ and LSFL ⊥ and their periods are investigated for a range of electron density values at various laser intensities based on Sipe^8,19^ and SP^58^ theories, respectively. Finally, a Two Temperature Model (TTM)^59^ coupled with a hydrodynamical module^10,11^ is used to describe energy transfer between the electron and the lattice systems and surface modification processes assuming that surface patterning requires a phase transformation and melting of the heated material.

## Results and Discussion

In this Section, the theoretical model that describes the electron excitation following irradiation of SiO_2_ with mid-IR pulses is presented. A more detailed presentation of the mathematical model is provided in the Supplementary material. Furthermore, the fundamental mechanisms of surface modification and the evaluation of the surface pattern periodicities are described through a multiscale theoretical model.

### Photoionisation processes

**Figure 1:**
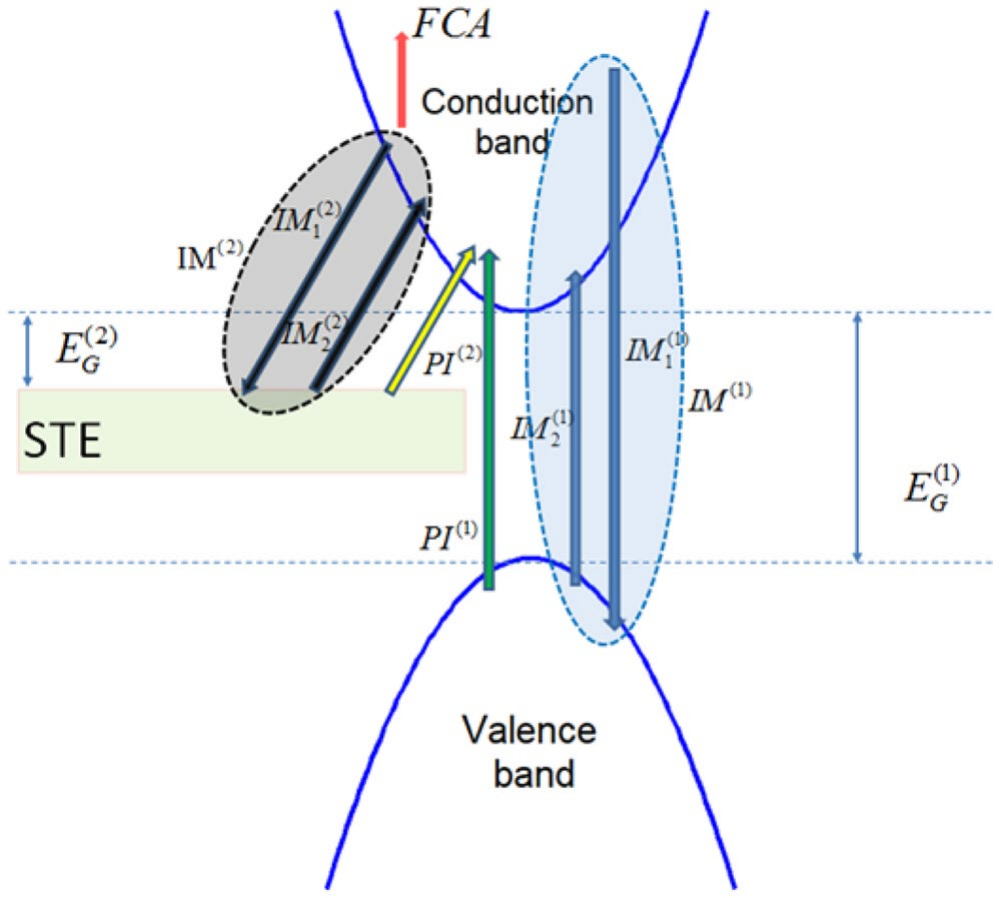
Excitation processes in fused silica: *Green* and *yellow* arrows indicate photoionisation mechanism (*PI*^(1)^ and *PI*^(2)^) from VB and self-trapped exciton (STE) levels, respectively. The position of the STE ‘box’ simply illustrates graphically that the STE band lies between VB and CB. *Blue* region corresponds to the impact ionisation process *IM*^(1)^ from CB to VB [*blue* down arrow indicates collision of highly energetic electrons in CB with electrons in VB $(I{M}_{1}^{(1)})$ while *blue* up arrow indicates transfer of the latter into CB $(I{M}_{2}^{(1)})$]. Similarly, *gray* region corresponds to the impact ionisation ($I{M}^{(2)})$ from CB to STE [*black* down arrow indicates collision of highly energetic electrons in CB with electrons in STE $(I{M}_{1}^{(2)})$ while *black* up arrow indicates transfer of the latter into the CB)]. ${E}_{G}^{(1)}$ (or ${E}_{G}^{(2)}$) stand for the band gaps between top of VB (or STE) and bottom of CB. *Red* arrow corresponds to inverse bremsstrahlung (free carrier absorption, FCA).

In the absence of defects, the absorption of light in dielectrics and photoionisation (PI) is a nonlinear process because a single photon does not have enough energy to excite electrons from the valence (VB) to the conduction band (CB). In the presence of intense laser fields, both multiphoton (MPI) and tunneling ionisation (TI) can occur in different regimes depending on the laser intensity values^50,60^. A detailed description of the ionisation processes is illustrated in Fig. 1. The free electrons in CB generated through PI operate as ‘seeds’ for a dominant excitation process, the impact ionisation^50^. The latter can occur once the conduction band electrons have sufficiently high energy; a part of their energy may be transferred to bound electrons in VB by collision (process $I{M}_{1}^{(1)}$ in Fig. 1) resulting in free electrons at the bottom of CB (process $I{M}_{2}^{(1)}$ in Fig. 1). These free electrons will subsequently experience the same process as discussed above, and produce more electrons in CB by exciting electrons from VB. On the other hand, relaxation processes lead to the production of metastable STE states^61^. In Fig. 1, STE states are illustrated as centres/defects situated in an energy region ${E}_{G}^{(2)}$ = 6 eV^49^ below the minimum of CB (smaller than the band gap of fused silica, ${E}_{G}^{(1)}$ = 9 eV). Trapping time of electrons in STE states is taken to be ${\tau }_{tr}$ ~ 150 fs^62^. If the laser pulse duration is *longer* than ${\tau }_{tr}$ and given that STE decay time is of the range of some hundreds of picoseconds^63,64^, excitation through photoionisation ($P{I}^{(2)}$) and impact ionisation ($I{M}^{(2)}$ through sequential processes $I{M}_{1}^{(2)}$ and $I{M}_{2}^{(2)}$ similar to those for $I{M}^{(1)}$ in Fig. 1) of STE states are possible^49,61^. Furthermore, electrons in CB can further absorb energy from the laser photons through a linear process, the free carrier absorption (denoted by FCA label in Fig. 1)^50^, and move to higher states in CB.

**Figure 2:**
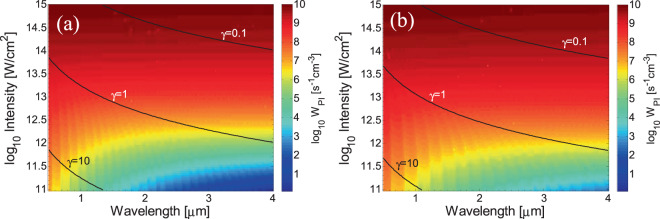
Photoionisation rates at various wavelengths and laser (peak) intensities assuming excitation of electrons from VB (**a**) and STE (**b**). The *black* lines define regions in which the Keldysh parameter *γ* attains values *γ* = 0.1, 1, 10.

**Figure 3:**
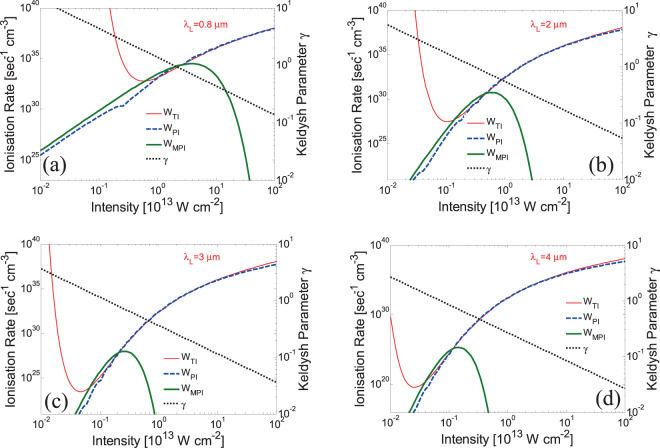
Photoionisation rates as a function of the laser (peak) intensity. Intensities at which multiphoton, tunneling and combined photoionisation dominate depending of the Keldysh number are illustrated. Results are shown for *λ*_*L*_ = 0.8 μm, 2 μm, 3 μm, 4 μm.

As stated above, PI depends on the laser intensity, however, MPI and TI are efficient in different regimes of the intensity spectrum. In Fig. 2, the photoionisation rate *W*_*PI*_ is computed for (peak) intensities *I* in the range between 10^11^ W/cm^2^ and 10^15^ W/cm^2^ and for laser wavelengths *λ*_*L*_ = 0.5–4 μm. Figure 2a,b describe photoionisation both of VB and STE electrons. The Keldysh parameter, *γ* ~ 1/($\sqrt{I}$*λ*_*L*_), indicates the regime in which each of the photoionisation components, MPI and TI dominates^60^. It is evident that at longer wavelengths and large intensities, TI dominates (*γ*≪1) while at shorter wavelengths and small intensities, MPI is the main contributor to PI (*γ*≫1). By contrast, an intermediate regime in which both TI and MPI coexist is illustrated in a region between *γ* = 0.1 and *γ* = 10. Previous studies demonstrated that the Keldysh approach to distinguish between the extremes of two different descriptions of ionization is valid in the mid-IR spectral region^42^. In Fig. 2, it is shown that pronounced ridges occur at low intensities (Fig. 2) for various wavelengths which are justified by the different number of photon energies required for MPI to ionise the material. On the other hand, the wavelength dependence disappears as *γ* moves to regimes where TI dominates which occurs also at smaller wavelengths^41,65^. The approximating values for TI and MPI as a function of the laser intensity at various wavelengths are illustrated in Fig. 3. To evaluate the regime where TI or MPI become more efficient in the PI process, the Keldysh parameter is also displayed. Results for three wavelengths in the mid-IR spectral region (*λ*_*L*_ = 2 μm, 3 μm, 4 μm) are compared with calculations for *λ*_*L*_ = 800 nm. It is evident that for large intensities, the Keldysh approximation for MPI provides an underestimation of PI rates while for lower values of *I* an overestimated TI is revealed. Interestingly, as the laser wavelength increases, the threshold value of *γ* at which MPI is less efficient than TI decreases. Furthermore, while at *λ*_*L*_ = 800 nm TI appears to become a dominant ionisation process at *γ* ~ 1, this behaviour occurs at even smaller values of *γ* (<0.1) at longer wavelengths (Fig. 3c,d).

### Electron excitation

**Figure 4:**
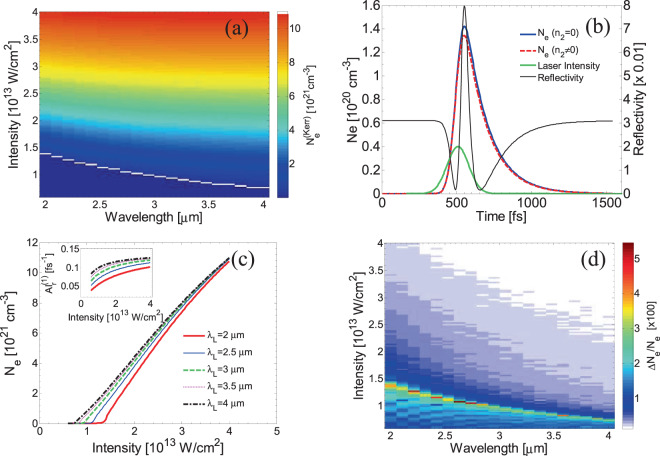
(**a**) MVED at various wavelengths and laser (peak) intensities (*white* line defines OBT). (**b**) Electron density evolution for *λ*_*L*_ = 2.6 μm and *I* = 1.26 × 10^13^ W/cm^2^ with and without Kerr effect. Laser pulse is shown in arbitrary units (*green* line). (**c**) MVED dependence on laser intensity (Results are shown for *λ*_*L*_ = 2 μm, 2.5 μm, 3 μm, 3.5 m and 4 μm). Inset illustrates the influence of impact ionisation. (**d**) Percentage change of MVED if Kerr effect is considered ($\Delta {N}_{e}=({N}_{e}-{N}_{e}^{(Kerr)})$). (*τ*_*p*_ = 170 fs).

To describe the free electron dynamics following excitation with ultrashort pulsed lasers, two single rate equations are used to account for the temporal evolution of the excited electrons from both VB and STE. Based on the scheme of the underlying physical mechanisms for photoionisation described in Fig. 1 and assuming laser pulse durations longer than *τ*_*r*_, excitation of STE to the CB is also considered. It is noted that the proposed model ignores the fact that only electrons in the CB with a sufficiently high kinetic energy are capable to participate in impact ionisation processes and enable electrons in the VB to overcome the ionisation potential. Revised models to remove this overestimation of the induced impact ionisation (with^11^ or without^48,51^ the inclusion of STE states) has been presented in previous reports in which multiple rate equations were introduced^51^. Nevertheless, the model that is used in this work is based on the following simplified set of two-rate equations (TRE) which have also been used in previous works at lower wavelengths^49,52,62,66^

1 $$\begin{array}{rcl}\frac{d{N}_{e}}{dt} & = & \frac{{N}_{v}-{N}_{e}}{{N}_{v}}({W}_{PI}^{(1)}+{N}_{e}A{I}_{r}^{(1)})+\frac{{N}_{STE}}{{N}_{v}}({W}_{PI}^{(2)}+{N}_{e}A{I}_{r}^{(2)})-\frac{{N}_{e}}{{\tau }_{tr}}\\ \frac{d{N}_{STE}}{dt} & = & \frac{{N}_{e}}{{\tau }_{tr}}-\frac{{N}_{STE}}{{N}_{v}}({W}_{PI}^{(2)}+{N}_{e}A{I}_{r}^{(1)})\end{array}$$

where $A{I}_{r}^{(1)}$ and $A{I}_{r}^{(2)}$ are the (usually termed^50,67^) avalache ionisation rate coefficients for the impact ionisation $I{M}^{(1)}$and $I{M}^{(2)}$ processes, *N*_*V*_ = 2.2 × 10^22^ cm^−3^ ^49^ corresponds to the atomic density of the unperturbed material while_*e*_ *N*_*e*_ and *N*_*STE*_ denote the free and STE electron densities, respectively. Finally, the photoionisation rates ${W}_{PI}^{(1)}$ and ${W}_{PI}^{(2)}$ include both multiphoton and tunneling ionisation processes; as noted in the previous paragraphs, each one of the multiphoton and tunneling ionisation processes become more efficient in different regimes based on the value of *γ*^49,60^. Maximum values of the electron densities (MVED) as a function of the laser wavelength and intensity are illustrated in Fig. 4a. It is noted that results for MVED include contribution from nonlinearities in the refractive i*n*dex *n* due to Kerr effect (denoted with ${N}_{e}^{(Kerr)}$); the influence of Kerr effect is discussed in the next section. In this study, simulations were performed for laser pulse duration equal to *τ*_*p*_=170 fs at different peak intensities $(I=\frac{2\sqrt{ln2}J}{\sqrt{\pi }{\tau }_{p}})$^43^ where *J* stands for the energy fluence. In the Supplementary Material, simulation results for MVED have been obtained for different values of the pulse duration and fluence to highlight the influence of STE states for single shot irradiation. Similar investigation can be pursued for multi pulse simulated experiments.

The temporal variation of reflectivity for the irradiated material (Fig. 4b) resembles the theoretical calculations and experimental results at shorter wavelengths^53,68^. The pulse is switched on at *t* = 0 and reaches the peak at *t* = *3τ*_*p*_ (*green* line in Fig. 4b). During the pulse, the reflectivity initially drops while it, gradually, starts to increase when material undergoes a transition to a ‘metallic’ state; then, the reflectivity reaches a peak value near the end of the pulse before it starts falling again.

An important outcome of the simulations is the dependence of the MVED on the laser intensities at different *λ*_*L*_, shown in Fig. 4c. It can be observed that the maximum values of the produced electrons for the same laser intensity rises at increasing wavelength. This is a result that has been observed experimentally in silicon^67^ at lower wavelengths while it has also been predicted for irradiation of silicon with mid-IR laser pulses^43^. The monotonicity is attributed to the increase of the strength of impact ionisation rate at increasing wavelength which is confirmed by the simulations (inset in Fig. 4c). In Fig. 4c, the impact ionisation rate illustrated for excitations from VB to CB (denoted by $A{I}_{r}^{(1)}$). Similar monotonicity occurs for impact related excitation processes for the STE states.

### Influence of kerr effect

To account for the Kerr effect, an intensity dependent variation of the dielectric constant $\Delta \varepsilon =2{n}_{0}{n}_{2}I+{({n}_{2}I)}^{2}$ is considered where *n*_*0*_ is the refractive index of the unexcited material and *n*_*2*_ is the Kerr coefficient which is related to the Kerr effect (see Supplementary Material). While extensive measurements of the nonlinear refractive index *n*_*2*_ exist for various types of materials in the visible and near-infrared (IR) wavelengths, few such measurements have been performed in the longer, spectroscopically important mid-IR regime. Experimental measurements for fused silica found in recent studies for *n*_*2*_ (equal to (1.94 ± 0.19) × 10^−16^ cm^2^/W and (2.065 ± 0.23) × 10^−16^ cm^2^/W for *λ*_*L*_ = 2.3 μm and 3.5 μm, respectively^69^), indicate a practically constant value for the Kerr coefficient. In principle, *n*_*2*_ for fused silica in the mid-IR spectral region appears to be 100 times smaller than the experimentally measured value for silicon^43^. The *white* line in Fig. 4a shows the intensity thresholds (i.e. optical breakdown threshold OBT) for which the simulated *N*_*e*_ exceeds the critical value ${N}_{e}^{cr}$(${\rm{i}}.\,{\rm{e}}.\,{N}_{e}^{cr}\equiv 4{\pi }^{2}{c}^{2}{m}_{e}{\varepsilon }_{0}/({\lambda }_{L}^{2}{e}^{2})$; ${N}_{e}^{cr}$ corresponds to the free electron density at which the plasma oscillation frequency is equal to the laser frequency, where *c* is the speed of light, *m*_*e*_ stands for the electron mass, *e* is the electron charge and ${\varepsilon }_{0}$ is the permittivity of vacuum). ${N}_{e}^{cr}$ is, often, associated with the induced damage on the material following exposure to intense heating, however, as it will be explained later in the text, a thermal criterion will be used in this work to describe damage^62^. To evaluate the influence of Kerr effect in the electron excitation, a series of simulations were performed to highlight the contribution of the Kerr effect at different intensities and wavelengths. In Fig. 4b, simulations for *I* = 1.26 × 10^13^ W/cm^2^ and *λ*_*L*_ = 2.6 μm, show that the maximum electron density lies higher than the MVED if Kerr effect is assumed. Similar conclusions follow at different wavelengths and intensities (Fig. 4d). Moreover, if Kerr effect is taken into account, the refractive index which is also a measure of reflectivity, increases as the intensity becomes higher due to the nonlinear part in the expression for *n* (for small intensity values). As a result, an enhanced reflectivity is produced which leads to a lower energy absorption from the material and therefore a lower excited electron density ${N}_{e}^{(Kerr)}$ (Fig. 4b).

Relevant simulations for MVED at wavelengths between *λ*_*L*_=2 μm and 4 μm and intensities up to *I* = 4 × 10^13^ W/cm^2^ are illustrated in Fig. 4d where a maximum 5% increase of the MVED is calculated. Interestingly, at a given wavelength, as the intensity increases there is an initially increase of the discrepancy which reaches the maximum value at around the intensity that yields ${\rm{MVED}}={N}_{e}^{cr}$, before it starts falling at larger values of *I* (Fig. 4d). The rising trend can be attributed to the calculated reflectivity and the resulting absorbed energy; at low intensities, reflectivity is small (Fig. 4b) that gives rise to larger differences, if Kerr effect is considered. By contrast, at higher intensities, the Kerr polarizability of the medium rapidly ceases to exist (the band structure is being progressively destroyed) due to ongoing ionisation; thus, the response of the medium is strongly dominated by the behaviour of the free electron-hole plasma which is formed during the pulse. This is followed by an increase of the reflectivity when the free electron density becomes higher than the critical density while it leads to a gradual drop of the electron density. The fact that the intensity values for which the difference $({N}_{e}-{N}_{e}^{(Kerr)})/{N}_{e}$ becomes maximum appear to coincide with OBT (Fig. 4a,d) requires more investigation. Although, the agreement does not appear to be coincidental, a deeper exploration of the role and interplay of various excitation processes should further analysed. Nevertheless, it is evident that a direct comparison with Kerr effect is not convincing as the expression of ${N}_{e}^{cr}$ is independent of the refractive index.

### LIPSS formation

**Figure 5:**
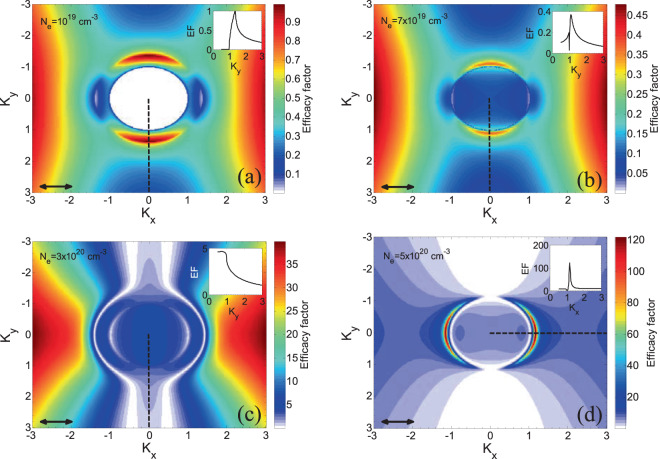
Efficacy factor maps for (**a**) *N*_*e*_ = 10^19^ cm^−3^, (**b**) *N*_*e*_ = 7 × 10^19^ cm^−3^, (**c**) *N*_*e*_ = 3 × 10^20^ cm^−3^ and (**d**) *N*_*e*_ = 5 × 10^20^ cm^−3^ (*λ*_*L*_ = 2.6 μm). Inset shows maps across the *black* dashed lines. *Black* double-ended arrows indicate polarisation direction.

**Figure 6:**
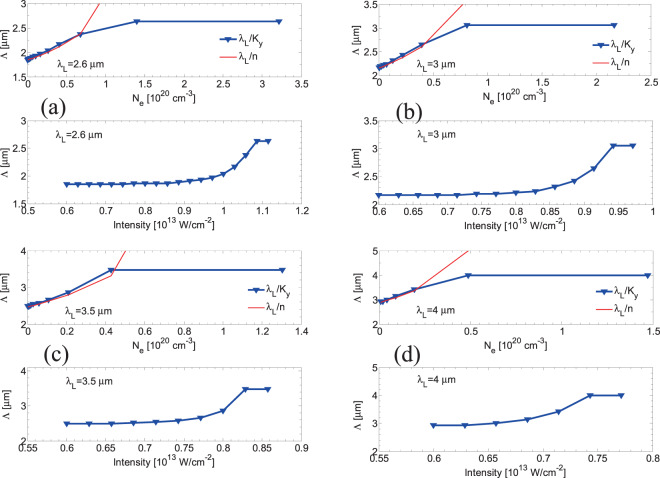
Calculated periodicities of parallel subwavelength LIPSS as a function of electron densities and laser (peak) intensities (*λ*_*L*_ = 2.6 μm, 3 μm, 3.5 μm, and 4 μm) (*τ*_*p*_ = 170 fs).

One of the predominant advantages of elucidating the interrelation of the processes taking place in SiO_2_ following irradiation with femtosecond laser pulses is that it can open unique ways for optimising and controlling patterning methodologies. It is known that energy release into the lattice depends to a large extent on the capability of the irradiated material to transfer electronic excitations into vibrational modes. The aforementioned model (Eq. 1) is aimed to enable determination of electron excitation levels and efficient description of electron dynamics which are both correlated with induced morphological changes on the surface or volume of the irradiated material. As explained in the introductory section of this work, LIPSS is one important type of surface patterns which is of paramount importance to a wide range of applications. To describe the formation of LIPSS, the electron density values and surface corrugation features are used to determine the orientation as well as the periodicity of LIPSS. In a similar fashion, as the procedure followed for irradiation with IR pulses^53^, a distinction is made about the conditions for formation of LSFLǁ and LSFL ⊥. Currently, the most widely accepted theory of LSFL is based on the interference of the incident laser beam with some form of a surface-scattered electromagnetic wave (SP waves could also be included if appropriate conditions are satisfied^58^). To correlate *N*_*e*_ with possible formation of laser-induced surface structures, the inhomogeneous energy deposition into the irradiated material is computed through the calculation of the product $\eta (\mathop{K}\limits^{\rightharpoonup },\,\overrightarrow{{k}_{i}})\times |b(\mathop{K}\limits^{\rightharpoonup })|$ as described in the model of Sipe^19^. In the above expression, *η* represents the efficacy with which a surface roughness at the wave vector $\mathop{K}\limits^{\rightharpoonup }$ (i.e., normalised wavevector $|\mathop{K}\limits^{\rightharpoonup }|$ =${\lambda }_{L}/\varLambda$, where $\varLambda$ stands for the predicted structural periodicity) induces inhomogeneous radiation absorption, $\overrightarrow{{k}_{i}}$ is the component of the wave vector of the incident laser beam on the material’s surface plane and *b* represents a measure of the amplitude of the surface roughness at $\mathop{K}\limits^{\rightharpoonup }$. In principle, surface roughness is considered to be represented by spherically shaped islands and standard values from Sipe’s theory for the ‘shape’ (*s* = 0.4) and the ‘filling’ (*f* = 0.1) factors^18,19,53^ are assumed. In this work, linearly polarised laser beams are used and according to Sipe’s theory, LIPSS are formed where *η*-maps exhibits sharp features (maxima or minima). Simulation results are illustrated in Fig. 5a–c at *λ*_*L*_ = 2.6 μm for some representative values of the carrier densities 10^19^ cm^−3^, 7 × 10^19^ cm^−3^ and 3 × 10^20^ cm^−3^. It is shown that sharp points of *η* appear along the ${K}_{y}(\,=\,{\lambda }_{L}/{\varLambda }_{y})$ axis (*black* dashed lines as shown in the insets) which indicates that LSFL are oriented *parallel* to the laser polarisation vector. Furthermore, as shown in the insets, the positions of the sharp features along *K*_*y*_ yield the periodicities of the periodic structures to be equal to ${\varLambda }_{y}$ = 1.9 μm, 2.38 μm and 2.58 μm (i.e. through the expression ${\varLambda }_{y}={K}_{y}/{\lambda }_{L}$), respectively. Therefore, Sipe’s theory is capable to predict efficiently both the orientation and periodicities of LSFLǁ. By contrast, for *N*_*e*_ = 5 × 10^20^ cm^−3^, production of LSFLǁ disappear as no structures with sharp features along *K*_*y*_ are predicted; by contrast, the *η*-map shows (Fig. 5d) that another type of structures (termed LSFL ⊥) is produced that are aligned *perpendicularly* to the laser polarisation. Simulation results illustrated in Fig. 5d indicate that the electron density plays a significant role towards switching the orientation of the induced periodic structures from LSFLǁ to LSFL ⊥. Similar conclusions were drawn at lower wavelengths, however, for remarkably higher values of *N*_*e*_^53^. The fact that slightly different electron densities result in a switch of orientation of the LIPSS by 90 degrees is a consequence of the different intensity values and absorption levels. This is an interesting outcome as it can be regarded as a saturation effect which can be employed in laser-based patterning techniques to produce either type of LIPSS in a controlled way by modulating the laser intensity (Fig. 6).

Simulations were also conducted to derive a correlation between the electron densities, the laser intensities and the periodicities for LSFLǁ for four values of the laser wavelength *λ*_*L*_ = 2.6 μm, 3 μm, 3.5 μm and 4 μm (Fig. 6). In regard to the periodicity dependence on the electron density, results show that at relatively low densities the simulated predictions for ${\varLambda }_{y}$ scale as ${\lambda }_{L}/n$ (*red* line in Fig. 6); by contrast, at larger *N*_*e*_, the expression ${\lambda }_{L}/n$cannot be used to calculate the periodicities of LSFLǁ. Similar conclusions were also drawn at lower wavelengths^53^. Furthermore, ripple periodicities saturate to a value close to *λ*_*L*_ at higher excitation levels and higher intensities.

**Figure 7:**
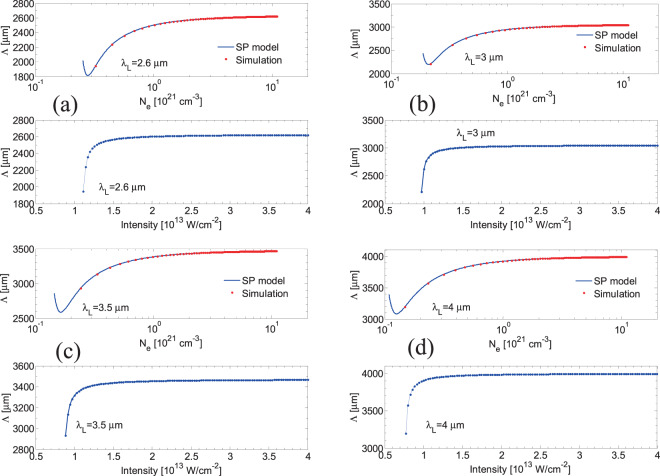
Calculated surface plasmon wavelengths as a function of electron densities and laser (peak) intensities (*λ*_*L*_ = 2.6 μm, 3 μm, 3.5 μm, and 4 μm) (*τ*_*p*_=170 fs).

In regard to the formation of LSFL ⊥, besides Sipe’s theory based on the interference of the incident light with the far-field of rough non-metallic or metallic surfaces^22^, an alternative approach assuming SP excitation will be used to correlate the simulated electron density values and the predicted frequencies of the periodic structures (a similar scenario was considered^53^ to explain the observed LSFL ⊥ structures at very short pulses ~5 fs^56^). This mechanism has been widely proposed for metals^1,13,14,24^ and semiconductors^10,33^, however, LSFL ⊥ formation *based on the excitation of SP in dielectrics* represents a yet unexplored field. According to the SP-model, the calculated periodicity is provided by the expression $\varLambda ={\lambda }_{L}/Re\sqrt{\frac{\varepsilon }{\varepsilon +1}}$^10,43^ for irradiation in vacuum which is approximately correct for very small number of pulses (*NP*)^13^. Results in Fig. 7 illustrate the computed values of *Λ* as a function of *N*_*e*_, both for the maximum electron densities calculated in this work (*red* dots) and for the range of values of *N*_*e*_ that are necessary to satisfy the condition for SP excitation (i.e. *Re(ε)* < −*1*)^43,58^. Results are also used to derive the correlation between the intensity values that give rise to SP excitation and therefore LSFL ⊥ periodic structures (Fig. 7). Simulations were performed for four values of the laser wavelength *λ*_*L*_ = 2.6 μm, 3 μm, 3.5 μm and 4 μm and results demonstrate a drop of *N*_*e*_ and *I* required to produce ripples at increasing laser wavelength.

The above paragraphs correlate the periodic features of LIPSS (periodicity and orientation) with the corrugation of the surface and the induced excited electron densities. Nevertheless, to provide a detailed description of LIPSS formation, a multiscale description of all physical processes that include energy absorption, electron excitation, transfer of heat from the electron to the lattice system, phase transitions and surface modification processes is required. More specifically, the following TTM (Eq. 2) is used to describe the increase of the electron temperature after excitation as well as the relaxation process and lattice temperature change following energy transfer from the electron system to the lattice

2 $$\begin{array}{rcl}{C}_{e}\frac{d{T}_{e}}{dt} & = & \overrightarrow{\nabla }({k}_{e}\overrightarrow{\nabla }{T}_{e})-g({T}_{e}-{T}_{L})+S\\ {C}_{L}\frac{d{T}_{L}}{dt} & = & \overrightarrow{\nabla }({k}_{L}\overrightarrow{\nabla }{T}_{L})+g({T}_{e}-{T}_{L})\end{array}$$

where *C*_*e*_ and *C*_*L*_ stand for the heat capacities of the electron and lattice subsystems, respectively while *T*_*e*_ and *T*_*L*_ are the temperatures of the two systems. On the other hand, *k*_*e*_ (*k*_*L*_) correspond to the electron (lattice) conductivity, *g* is the electron-phonon coupling and *S* is a source term that describes the average energies of the particles which are excited with the laser beam. A more detailed description of Eq. 2 is provided in the Supplementary Material. It is noted that the employment of Eq. 2 is based on the consideration that the laser pulse duration is long enough to assume an instantaneous thermalisation of the electron system through electron-electron scattering processes. By contrast, for very short pulses (<100 fs), a more thorough investigation is required to describe both the electron excitation, ultrafast dynamics and relaxation processes, however, this is beyond the scope of the present study.

To model the surface modification, it is assumed that the laser fluence is sufficiently high to result in a phase transition from solid to liquid phase and upon resolidification, a nonflat relief is induced on the surface of the material. Depending on the laser intensity, mass removal is also possible if the material is heated above a critical temperature (~*T*_*L*_ > $1.8\times {T}_{boiling}$ for SiO_2_ where ${T}_{boiling}=2270\,{\rm{K}}$^70^; the choice of the critical temperature is explained in the Supplementary Material). The movement of a material in the molten phase is given by the following Navier-Stokes equations (NSE) which describes the dynamics of an uncompressible fluid

3 $${\rho }_{0}\left(\frac{\partial \overrightarrow{u}}{\partial t}+\overrightarrow{u}\cdot \overrightarrow{\nabla }\overrightarrow{u}\right)=\overrightarrow{\nabla }\cdot (-P+\mu (\overrightarrow{\nabla }\overrightarrow{u})+\mu {(\overrightarrow{\nabla }\overrightarrow{u})}^{T})$$

where ${\rho }_{0}$ and $\mu$ stand for the density and viscosity of molten SiO_2_, while *P* and $\overrightarrow{u}$ are the pressure and velocity of the fluid, respectively. A more detailed description of the fluid dynamics module and numerical solution of NSE are provided in the Supplementary Material). In Eq. 3, superscript *T* denotes the transpose of the vector $\overrightarrow{\nabla }\overrightarrow{u}$^10^.

A multiple pulse irradiation scheme is required to derive the formation of a periodic relief^10,11^ through the solution of Eqs. 1–3. More specifically, LIPSS are formed in the following steps (an analytical description of the numerical solution is provided in the Supplementary Material)

- the first pulse irradiates a flat surface and it is assumed to melt/ablate (depending on the laser intensity) part of the material leading to the formation of a crater and humps at the edges on the surface of the heated zone due to mass displacement^10,11^. As the first pulse irradiates a flat surface with no corrugations, formation of periodic structures is not expected to occur^19^. It is noted that due to the axial symmetry of a Gaussian beam, for *NP* = 1, Eqs. 1–3 can be solved in 2D (*r* and *Z* where *r* is the horizontal distance from the position of the maximum energy absorption while *Z* stands for the vertical distance from the surface)^10^,
- the second pulse irradiates the previously formed profile and therefore the spatial symmetry breaks; as a result, a 2D modelling can no longer be used. Based on the discussion in the beginning of the section, the coupling of the electric field of the incident beam with the induced surface scattered wave produces a nonuniform, periodic distribution of the absorbed energy (of periodicity and orientation determined by the density of excited carriers and the efficacy factor). More specifically, the *η*-map dictates the propagation direction of the spatial modulation of the deposited energy (Fig. 5) with a *N*_*e*_-dependent periodicity ${\varLambda }_{y}$ (Fig. 6)^8,21^. In the present model, the induced spatial modulation of the absorbed energy is introduced as a sinusoidal function of periodicity ${\varLambda }_{y}$ in Eqs. 1 and 2 ^13^ (this approach allows modelling formation of LSFL||; on the other hand, at higher excitation levels when LSFL ⊥ structures are produced, as explained above, the periodicity can be determined either by the use of the efficacy factor map or the SP-model). The periodic variation of the absorbed energy leads to a periodic excited electron density distribution which is simulated with Eq. 1. It is noted, though, that the computation of the amount of the absorbed energy at each position requires the evaluation of the energy deposition on a curved surface^10,13^. Therefore, appropriate computational schemes have been developed to compute the absorbed energy on each point of the curved surface^10^. The spatially modulated electron energy distribution is transferred to the lattice system (through Eq. 2) and subsequently, upon phase transition (Eq. 3), fluid transport and resolidification processes, LIPSS are formed.
- In an iterative fashion, each subsequent pulse irradiates a periodic pattern that has been created in the previous step; it is noted that the depth of the surface profile is increased with increasing *NP*^9,13^ while the periodicity of the induced pattern is always determined by the carrier density as mentioned in the previous section. The temporal separation between subsequent pulses is long enough to ensure that each pulse irradiates material in solid phase. These multiscale mechanisms have been simulated and observed in other irradiation conditions and at lower wavelengths in previous reports^10–14,18,22^.

**Figure 8:**
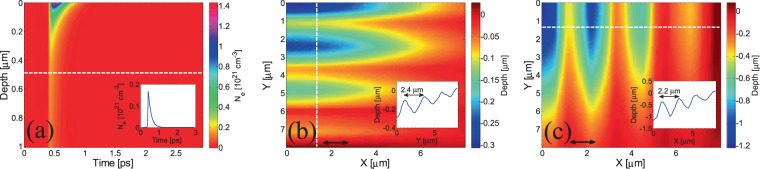
(**a**) Evolution of the spatial distribution of *N*_*e*_ along *z*-axis at position where energy deposition is higher for *NP* = 1 (for *I* = 1.4 × 10^13^ W/cm^2^), (dashed *white* line corresponds to the maximum depth for which OBT occurs while inset shows the temporal profile of *N*_*e*_ along the dashed line). (**b**) Top view of Parallel and (**c**) Perpendicular to laser polarisation LSFL for laser (peak) intensities *I* = 1.06 × 10^13^ W/cm^2^ and *I* = 1.4 × 10^13^ W/cm^2^, for *NP* = 3 and *NP* = 10, respectively. *Black* double-ended arrows indicate polarisation direction. (insets in (**b**,**c**) show the depth profiles along the dashed lines) (*τ*_*p*_ = 170 fs). Figures (**b**,**c**) illustrate a quadrant of the affected zone (top view) (*λ*_*L*_ = 2.6 μm).

As an example, results for a multipulse simulated experiment are shown in Fig. 8. The spatio-temporal evolution of the electron density following irradiation of fused silica with one pulse (*NP* = 1) of wavelength *λ*_*L*_ = 2.6 μm and a laser peak intensity *I* = 1.4 × 10^13^ W/cm^2^ is displayed in Fig. 8a. Assuming that the electron density which leads to optical breakdown is equal to ${N}_{e}^{cr}$ = 1.6561 × 10^20^ cm^−3^ for irradiation at this wavelength, a region for which ${N}_{e} > {N}_{e}^{cr}$ extends to ~490 nm (dashed line in Fig. 8a). The inset in Fig. 8a illustrates the temporal evolution of *N*_*e*_ at 490 nm. The value of ${N}_{e}^{cr}$ has been usually attributed to the onset of damage^62^ in spite of the fact that mass removal or melting occurs at longer times and after sufficiently high energy is transferred to the lattice system. By contrast, in this work, a thermal criterion is used (i.e. damage occurs when lattice temperature exceeds the melting point of the material) that constitutes a more precise estimation of the damage^11,12,49^. It is emphasised that the consideration of the peak intensity in the calculations was to compute the maximum depth of the heated region. Given the (spatially) Gaussian profile of the beam, it is evident that the maximum depth occurs where the absorbed energy is maximum. Figure 8b,c illustrates a top view (quadrant) of the surface profile for *λ*_*L*_ = 2.6 μm and laser peak intensities of 1.06 × 10^13^ W/cm^2^ and 1.4 × 10^13^ W/cm^2^, respectively, for *NP* = 3 and *NP* = 10 respectively. Based on the above discussion, the maximum depth occurs at *X* = *Y* = *0* (the position of the Gaussian intensity peak). The FWHM diameter of the laser spot is equal to 30 μm. The two intensities correspond to energies that lead to the formation of LSFLǁ and LSFL ⊥, respectively. The insets in Fig. 8b,c illustrate the depth profile along the *white* dashed line which shows a pronounced periodic spatial profile. The predicted periodicities for the two patterns are 2.4 μm and 2.2 μm.

A fundamental question that rises about the calculation of the induced periodicities as a function of the irradiation dose is whether the models centred on (i) Sipe’s or (ii) SP-based theories are valid with increasing *NP*. With respect to the former model, it is already known that one of the limitations of the efficacy factor-based theory is the neglect of the so-called ‘feedback mechanism’ which is very important to calculate the evolution of the periodicity of the induced periodic structures^19^. In the current work, firstly, simulations for *NP* = 2 for laser peak intensities of 1.06 × 10^13^ W/cm^2^ were performed assuming the conventional Sipe’s theory (i.e. *NP* = 1 corresponds to the irradiation of a flat profile and therefore Sipe’s theory cannot be used). The application of the multiscale model developed in this work and the use of Sipe’s theory predict a maximum value of the carrier density equal to ~0.7 × 10^21^ cm^−3^ that yields a periodicity ~2.35 μm. It is assumed that similar values for ‘shape’ and ‘filling’ parameters (*s* and *f*, respectively*)* can be used for *NP* = 3 assuming a relatively similar corrugation and comparable carrier density; hence, a computed value equal to approximately ~2.4 μm is derived for *NP* = 3 (Fig. 8b). In all cases, LSFLǁ are produced (as shown in Fig. 5a–c). On the other hand, as the depth of the surface pattern increases and its corrugation changes, a calculation of interpulse evolution of LSFLǁ periodicities with the employment of Sipe’s theory becomes problematic.

By contrast, in Fig. 8c, the periodic profile is illustrated for *NP* = 10 following irradiation with 1.4 × 10^13^ W/cm^2^. Results in Fig. 7a indicate that, for an almost flat profile, this laser intensity yields a carrier density ~1.47 × 10^21^ cm^−3^ that leads to a SP periodicity equal to 2.55 μm. On the other hand, the computed value for the ripple periodicity for *NP* = 10 is equal to ~2.2 μm. The latter value differs from the one computed through the expression $\varLambda ={\lambda }_{L}/Re\sqrt{\frac{\varepsilon }{\varepsilon +1}}$ which holds for nearly flat surfaces as enhanced corrugation has proven to yield a shift to the SP resonance to smaller values of *Λ* at increasing *NP*; this is also shown in previous studies in which lower laser wavelengths were used to fabricate LSFL ⊥ in metals or semiconductors^9,10,13,23^. In contrast to electrodynamics simulations, mainly, based on Finite Difference Finite Domain Schemes (FDTD) used to correlate the induced LIPSS periodicities with a variable corrugation as a result of increase of the irradiation dose^9,22,48,71,72^, an alternative and approximating methodology was employed, in this work, to relate the SP wavelength with the produced maximum depth of the corrugated profile^13,14^ (i.e. which is linked with *NP*). The methodology was based on the spatial distribution of the electric field on a corrugated surface of particular periodicity and height and how continuity of the electromagnetic fields influences the features of the associated SP. The variation of the SP wavelengths (which also determines the LSFL ⊥ periodicity) as a function of *NP* are shown in the Supplementary Material. Although, this technique provided theoretical predictions that agreed sufficiently well with experimental results^14^, an FDTD-based analysis is mainly considered to represent a more accurate tool to describe the electrodynamical effects and evaluate periodicities of LSFL ⊥ and LSFL||.

The multiscale model used in this work showed the formation of LSFL|| and LSFL ⊥ while a transition from one type to another (considering the discussion about Fig. 5) can also be derived, however, the types of structures that are produced for small number of *NP* (i.e. formation of LSFL||) differs from results in previous combined theoretical simulations^22^ and experimental observations^53,73^ at *shorter* wavelengths. More specifically, previous studies showed that for small number of pulses and fluences, HSFL structures are formed on the surface of the material while LSFL|| are induced at greater depths. The mixture of the two types of structures is also witnessed in diffraction experiments. An increase of the fluence or higher irradiation dose leads to a removal of the HSFL structures while LSFL|| structures remain. Further irradiation leads to a transition from LSFL|| to LSFL ⊥. Certainly, a future revised version of the model including more precise electromagnetic simulations would allow to understand further LIPSS evolution with laser sources in the mid-IR spectral region.

It should be emphasised that, a more accurate and convincing conclusion about the validity of the aforementioned model and theoretical predictions to describe physical mechanisms in the mid-IR region for fused silica will be drawn if appropriately developed experimental protocols are introduced. To the best of our knowledge, there are no previous experimental reports that could provide a validation of the aforementioned model for SiO_2_ in that spectral region. However, employment of Eq. 1 (to compute electron excitation^49,50,52,62,70^), Sipe theory mechanisms (to evaluate LSFL periodicities^11,12,19,53,74^) and Eqs. 1–3 (to derive a multiscale description of the surface patterning mechanisms^11^ with experimental validation^12,53,74^) showed that the theoretical model presented in this study is capable to explain efficiently physical processes at *shorter* wavelengths.

Moreover, a question that is raised with respect to the frequencies of the induced periodic structures is the impact of STE. The theoretical model presented in this work (and results shown in the Supplementary Material) shows, firstly, that there is a conspicuous influence of the STE on the carrier densities that, in turn, affect the LIPSS periodicities. Previous experimental results related to the investigation of femtosecond diffraction dynamics of LIPSS on fused silica at lower wavelengths^73^, showed that STE are capable to influence the refractive index values and facilitate emergence of incubation effects. Therefore, similar experimental protocols are important for the comparison of results from the proposed STE-based model with experimental findings at longer wavelengths.

In conclusion, it is evident that the lack of experimental results might hinder the validation of the theoretical framework at *longer* wavelengths. An extension of the model used for semiconductors (i.e. Silicon^43^) to simulate damage thresholds was also recently validated by appropriate experimental protocols^38,43^. In regard to the limitations of the model, there are some yet unexplored issues that need to be addressed (i.e. investigation of behaviour in ablation conditions, consideration of formation of voids inside the material after repetitive irradiation, role of incubation effects^22^, STE and defects^75^, validity of the use of Eqs. 1 and 2 for very short pulses where a more precise quantum mechanical approach is required to describe ultrafast dynamics^76^, influence of ambipolar electron-hole plasma diffusion to damage thresholds^77^, etc.) before a complete picture of the physical processes with femtosecond mid-IR laser pulses is attained. Nevertheless, the model is aimed to set the basis for a description of the multiscale processes that lead to surface modification in the mid-IR spectral region through the evaluation of the impact of the long pulses on various fundamental processes. On other hand, apart from the importance of elucidating the underlying mechanisms from a physical point of view, a deeper understanding of the response of the material will allow a systematic novel surface engineering with strong mid-IR fields for advanced industrial applications.

## Conclusions

To summarise, it is known that while an extensive research has been conducted towards elucidating laser-induced growth of damage for irradiation of SiO_2_ with IR (or shorter) pulses, little is known about the effects of electron excitation with longer wavelength pulses. In this work, a detailed theoretical framework was presented for the first time that describes both the ultrafast dynamics and thermal response following irradiation of fused silica with ultrashort pulsed lasers in the mid-IR spectral region. The influence of nonlinearities in the refractive index, the ultrafast dynamics in a wide range of wavelengths and various intensities, as well as fundamentals of laser-based surface patterning, were investigated. There is no doubt that our theoretical approach requires validation and possibly further development before it can fully account for the physical processes taking place upon laser-material interaction in the mid-IR spectral region. Nevertheless, the predictions resulting from the above theoretical approach demonstrate that unravelling phenomena during such interaction can potentially set the basis for the development of new tools for a large range of mid-IR laser-based applications.

## Methods

### Computational method

Full details of the multiscale model, numerical solution of the model and the parameters used are presented in the Supplementary Material.

## Supplementary information

Supplementary information.
