# Supplementary material for: Ionisation processes and laser induced periodic surface structures in dielectrics with mid-infrared femtosecond laser pulses
Source: Sci Rep. 2020 May 26;10:8675. doi: 10.1038/s41598-020-65613-w (PMC7250856; doi:10.1038/s41598-020-65613-w)
Supplement: Supplementary file 1 — Supplementary information. [file 41598_2020_65613_MOESM1_ESM.pdf]

Supplementary Material to 'Ionisation processes and laser induced periodic surface structures in dielectrics with mid-infrared femtosecond laser pulses'

George D. Tsibidis[1,*], and Emmanuel Stratakis[1,2]

[1] Institute of Electronic Structure and Laser (IESL), Foundation for Research and Technology (FORTH), N. Plastira 100, Vassilika Vouton, 70013, Heraklion, Crete, Greece
[2] Department of Physics, University of Crete, 71003 Heraklion, Greece

Email: [*]tsibidis@iesl.forth.gr

I. THEORETICAL MODEL

A. Electron density rate equations

The absorption of light in transparent materials is a nonlinear process as a single photon does not have enough energy to excite electrons from the valence to the conduction band. Following nonlinear photo-ionisation (where both multiphoton and tunnelling ionisation can occur in different regimes depending on the laser intensity), impact ionisation and formation of STE lead to a variation of the electron densities. To calculate the laser absorption, the densities of the excited electron and the STE are computed by solving the following set of equations

$$\begin{aligned} \frac{dN_e}{dt} &= \frac{N_v-N_e}{N_v}\left(W_{PI}^{(1)}+N_eAI_r^{(1)}\right)+\frac{N_{STE}}{N_v}\left(W_{PI}^{(2)}+N_eAI_r^{(2)}\right)-\frac{N_e}{\tau_{tr}} \\ \frac{dN_{STE}}{dt} &= \frac{N_e}{\tau_{tr}}-\frac{N_{STE}}{N_v}\left(W_{PI}^{(2)}+N_eAI_r^{(1)}\right) \end{aligned} \tag{1}$$

where N_V=2.2×10^{22} cm^{-3} [1] corresponds to the atomic density of the unperturbed material while N_e and N_{STE} denote the free electron and STE electron densities, respectively. The last term in the first equation corresponds to free electron decay that is characterised by a time constant τ_{tr} (τ_{tr} ~150fs in fused silica) that leads to a decrease of the electron density. By contrast, $W_{PI}^{(i)}+N_eAI_r^{(i)}$ (i=1,2) correspond to the combined photo-ionisation $W_{PI}^{(i)}$ and impact ionisation; $AI_r^{(i)}$ stands for the (usually termed) avalanche ionisation rate coefficients [1,2] of the impact ionisation term that depends on which bandgap the electrons need to surpass ($E_G^{(1)}$ (=9eV) or $E_G^{(2)}$(=6eV)). The photoionisation rates are computed using the Keldysh formulation [2]

$$W^{(i)}_{PI}=\frac{2\omega}{9\pi}\left(\frac{m_r\omega_L}{\gamma_2\hbar}\right)^{3/2}\Theta(\gamma,x)\exp\left[-\pi\langle x+1\rangle\frac{K(\gamma_2)-E(\gamma_2)}{E(\gamma_1)}\right] \tag{2}$$

where $\gamma=\omega_L\sqrt{m_rE_G^{(i)}}/(e|\vec{E}|)$ is the Keldysh parameter for the band-gap $E_G^{(i)}$ and is dependent on the electron charge e, the frequency ω_L and the field $|\vec{E}|$ of the laser beam, the electron reduced mass $m_r=0.5m_e$ (m_e is the electron mass) and $\gamma_2=\gamma/\sqrt{1+\gamma^2}$ and $\gamma_1=\gamma_2/\gamma$. Furthermore, $\langle x+1\rangle$ stands for the integer part of

the number $x+1$, where $x=2E_G^{(i)}E(\gamma_1)/(\pi\gamma_2\hbar\omega_L)$ while K and E are the complete elliptic integrals of the first and second kind, respectively. Also,

$$\Theta(\gamma, x) = \sqrt{\frac{\pi}{2K(\gamma_1)}}\sum_{N=0}^{\infty}\exp\left[-N\pi\frac{K(\gamma_2)-E(\gamma_2)}{E(\gamma_1)}\right]\times\Phi\left[\sqrt{\frac{\pi^2\left(2\langle x+1\rangle-2x+N\right)}{K(\gamma_1)E(\gamma_1)}}\right] \tag{3}$$

where $\Phi(z)=\int_0^z exp\left(y^2-z^2\right)dy$. Finally, the coefficient $AI_r^{(i)}$ is given by the following expression

$$AI_r^{(i)} = \frac{e^2\tau_c I}{\left[c\varepsilon_0 n m_r\left(\omega_L^{\,2}\left(\tau_c\right)^2+1\right)\left(2-m_r/m_e\right)\right]}\frac{1}{E_G^{(i)*}} \tag{4}$$

where $E_G^{(i)*} = E_G^{(i)} + e^2\left|\vec{E}\right|^2/\left(4m_r\omega_L^{\,2}\right)$ is the effective band gap which takes into account the oscillation energy of the free electrons in the electric field, and the conservation of energy and the momentum during the collision between the free and bound electrons. Also, c is the speed of light, ε_0 stands for the vacuum permittivity, n is the refractive index of the material, while I is the peak intensity of the laser beam, respectively, and τ_c is the electron collision time.

In regard to the value of τ_c, there are many reports in which the collision time has been considered to be a constant or a varying parameter. More specifically, values of τ_c vary in a range between 0.1 fs and 10 fs [3] while in other studies values equal to 1.0 fs [4], 1.5 fs [5], 23.3 fs [6], 10 fs [7], 1.7 fs [8], 0.5 fs [9] have been reported. Thus, simulations with the above choices for τ_c yielded good agreement with experimental data.

On the other hand, there are other reports in which a more rigorous analysis was followed to compute the collision time: In Chimier et al [1], the contribution of electron-phonon, electron-electron, electron-neutral and electron-ion collision frequencies were used to compute τ_c. In other reports [10-12], the electron density distribution function was also considered to calculate the electron collision frequency and the electron collision time. A remarkable impact of the value of the collision frequency on observable quantities such as the damage threshold has been demonstrated with simulations in other materials (i.e. Silicon [13]) which emphasized the need for a precise evaluation. Nevertheless, in the absence of experimental results in the mid-IR, the aim of the present work was firstly to provide a consistent multiscale modelling approach in which some approximations have been made rather than to consider a more precise evaluation of the collision frequency that could be the subject of revised model. Therefore, the collision frequency is taken constant in this work. In regard to the value chosen in this manuscript, the motivation was based on the work of Burakov et al [5] in which the damping term $\omega_L\tau_c$=3 was used that resulted into a collision time equal to1.5 fs (for λ_L=800 nm). In the case of mid-IR, for laser wavelengths between 2 μm-4 μm, the collision time was calculated from the above expression yielding values for τ_c in the range [3.1fs, 6.4fs].

The dependence of the avalanche ionization rate coefficient $AI_r^{(i)}$ on the collision time to which carrier-carrier or carrier-ion scattering times (and dephasing oscillations) contribute [14,15] implies that a possible future revision of the model should include a term for impact ionization in which the collision time is a varying parameter; in relevant reports [14,15], an electron temperature dependent expression for τ_c was considered.

The photoionisation rates $W_{PI}^{(1)}$ and $W_{PI}^{(2)}$ include both multiphoton and tunneling ionisation processes that are provided from the following expressions

$$MPI^{(i)} = \frac{2\omega_L}{9\pi}\left(\frac{m_r\omega_L}{\hbar}\right)^{3/2}\Phi\left(\sqrt{2\left\langle\frac{E_G^{(i)}}{\hbar\omega_L}+1\right\rangle-2\frac{E_G^{(i)}}{\hbar\omega_L}}\right)\times\exp\left(2\left\langle\frac{E_G^{(i)}}{\hbar\omega_L}+1\right\rangle\left(1-\frac{1}{4\gamma^2}\right)\right)\left(\frac{1}{16\gamma^2}\right)^{\left\langle\frac{E_G^{(i)}}{\hbar\omega_L}+1\right\rangle} \tag{5}$$

$$TI^{(i)} = \frac{2E_G^{(i)}}{9\pi^2\hbar}\left(\frac{m_r E_G^{(i)}}{\hbar^2}\right)^{3/2}\left(\frac{e\hbar|\vec{E}|}{\sqrt{m_r}\left(E_G^{(i)}\right)^{3/2}}\right)^{5/2} \exp\left(-\frac{\pi E_G^{(i)}}{2\hbar\omega_L\gamma}\left(1-\frac{\gamma^2}{8}\right)\right) \quad (6)$$

The two ionisation processes become more efficient in different regimes; more specifically, tunneling ionisation becomes dominant for $\gamma<<1$ while multiphoton ionisation becomes more efficient for $\gamma>>1$ [2]. In contrast to the avalanche ionisation rate coefficient and impact ionisation, multiphoton and tunneling ionisation rates do not appear to be dependent on dephasing effects.

With respect to the intensity of the laser beam I, previous studies consider that the attenuation of the local laser intensity is determined by multiphoton ionisation and inverse bremsstrahlung (Free Carrier) absorption [9]. Nevertheless, the presence of STE states and the possibility of retransfer of carriers in these states back to the conduction band through multiphoton ionisation require modification of the spatial intensity distribution; therefore a revised 3D model (in Cartesian coordinates: $\vec{x}=(X,Y,Z)$) should include those contributions [1,16,17]

$$\begin{aligned} \frac{\partial I(t,\vec{x})}{\partial t} &= -N_{ph}^{(1)}\hbar\omega_L \frac{N_v - N_e}{N_v} PI^{(1)} - \alpha(N_e) I(t,\vec{x}) - N_{ph}^{(2)}\hbar\omega_L \frac{N_{STE}}{N_v} PI^{(2)} \\ I(t,X,Y,Z=0) &= (1-R(t,X,Y,Z=0)I_{peak} \exp\left(-4\log(2)\left(\frac{t-3\tau_p}{\tau_p}\right)^2\right)\exp\left(-\frac{X^2+Y^2}{(R_0)^2}\right) \end{aligned} \quad (7)$$

where $N_{ph}^{(i)}$ corresponds to the minimum number of photons necessary to be absorbed by an electron that is in the valence band (i=1) or the band where the STE states reside (i=2) to overcome the relevant energy gap and reach the conduction band. I_{peak} ($\equiv \frac{2J\sqrt{ln2}}{\sqrt{\pi}\tau_p}$) corresponds to the peak value of the laser intensity, J stands for the (peak) laser fluence, $R(t,x,y,z=0)$ stands for the reflectivity of the material while R_0 is the irradiation spot radius (R_0=15μm in our simulations). The second equation of Eq.7 gives the spatial distribution of the intensity profile in Cartesian coordinates (and on the surface of the irradiated material).

It is also noted that, for the sake of simplicity, it is assumed that the beam shape does not change upon propagation; thus, only attenuation losses are taken into account and no Kerr or plasma distortion to the pulse phase were considered. A more accurate description of the laser energy distribution has been presented in other studies, by solving Maxwell's equations [9] or by including the shape change and Kerr effect to evaluate damage in the bulk [5]. However, we believe that a more accurate expression for the intensity profile would not lead to substantially different surface effects.

B. Dielectric constant

The refractive index of fused silica when it is in an unexcited state is denoted by n_0 [18] (at I=0) ant it provided by the following expression

$$n_0 - 1 = \frac{0.6961663}{\lambda_L^2 - 0.0684043^2}\lambda_L^2 + \frac{0.4079426}{\lambda_L^2 - 0.1162414^2}\lambda_L^2 + \frac{0.8974794}{\lambda_L^2 - 9.896161^2}\lambda_L^2 \quad (8)$$

The expression $\varepsilon_{un} = n_0^2$ yields the dielectric constant of the unexcited material. Following irradiation of SiO_2 with femtosecond pulses, a temporally dependent density of excited carriers is produced that modify the dielectric constant of the material

$$\varepsilon' = 1+(\varepsilon_{un}-1)\left(1-\frac{N_e}{N_v}\right)-\frac{e_c{}^2 N_e}{m_r m_e \varepsilon_0 \omega_L{}^2}\frac{1}{\left(1+i\frac{1}{\omega_L \tau_c}\right)} \tag{9}$$

The reflectivity and free carrier absorption coefficients are given by the following expressions

$$\alpha_{FCA} = \frac{2\omega_L k}{c}$$
$$R = \frac{(1-n)^2+k^2}{(1+n)^2+k^2} \tag{10}$$

where k is the extinction coefficient of the material. The real part of the refractive index n is the sum of two terms, one (n_0) that corresponds the refractive index in an unexcited state and a second ($n_2 I$) that corresponds to the nonlinearities induced by Kerr effect (i.e. $n=n_0+n_2 I$). n_2 stands for the Kerr coefficient. On the other hand, the dielectric constant is given by the following expressions if the Kerr effect is taken into account [19]

$$\varepsilon = \varepsilon' + \Delta\varepsilon_{\mathrm{kerr}}$$
$$\Delta\varepsilon_{\mathrm{kerr}} = 2 n_0 n_2 I + (n_2 I)^2 \tag{11}$$

while $\varepsilon=(n+ik)^2$

C. Electron and lattice heat balance

To describe the morphological properties due to laser irradiation of the material, it is important to explore, firstly, the relaxation process and heat transfer from the ionised material to the lattice system. Due to the metallic character of the excited material, a TTM model can describe the spatio-temporal dependence of the temperatures T_e and T_L of the electron and lattice subsystems, respectively [20]

$$\begin{aligned} C_e \frac{dT_e}{dt} &= \vec{\nabla}(k_e \vec{\nabla} T_e) - g(T_e - T_L) + S \\ C_L \frac{dT_L}{dt} &= \vec{\nabla}(k_L \vec{\nabla} T_L) + g(T_e - T_L) \end{aligned} \tag{12}$$

The source term has been modified properly to take into account all quantities that contribute to the total electron energy balance. Hence, the complete expression for source term $S(\vec{x},t)$ is given by [1,11,17]

$$\begin{aligned} S(\vec{x},t) = {} & \left(N_{ph}^{(1)}\hbar\omega_L - E_G^{(1)}\right)\frac{N_v - N_e}{N_v} PI^{(1)} - E_G^{(1)} AI_r{}^{(1)} N_e \frac{N_v - N_e}{N_v} \\ & + \left(N_{ph}^{(2)}\hbar\omega_L - E_G^{(2)}\right)\frac{N_{STE}}{N_v} PI^{(2)} - E_G^{(2)} AI_r{}^{(2)} N_e \frac{N_{STE}}{N_v} \\ & + \alpha(N_e)\, I(t,\vec{x}) \\ & - \frac{3}{2} k_B T_e \frac{N_e}{\tau_r} - \frac{3}{2} k_B T_e \frac{dN_e}{dt} \end{aligned} \tag{13}$$

The source term includes terms that are related to the dynamics of the total energy of the electron system. Processes that are taken into account, are the photoionisation of electrons (first and third terms), impact ionisation (second and fourth terms). It is noted that excitation both from VB (first and second terms) and STE (third and fourth) electrons are considered. Finally, the fifth term stands for the free electron absorption (that leads to transition to higher states in the CB) while the last two terms correspond to the

energy balance due to electron density reduction (due to defect formation/trapping) and electron energy assuming carrier density variation [21]. Alternative expressions that incorporate average energies of electrons that are excited from STE states and exciton decay have been also proposed [11], however they have not been used in this work. The reason is that on the one hand, exciton decays are supposed to last very long (in the range of some hundreds of picoseconds [22,23]) compared to the timescales considered in the excitation process (and therefore their contribution is supposed to be insignificant); furthermore, the average energies of STE electrons contribution has been ignored in this study assuming it is not very significant compared that of the electron in CB. Nevertheless, a more precise investigation might be required in a future study by taking into account the role average energy of electrons in STE states in various laser conditions. We point out that a term that describes the divergence of the current of the carriers ($\vec{\nabla}\cdot\vec{J}$) has not been taken into account (it turns out that the consideration of particle transport of heat diffusion does not vary significantly quantities such as the damage threshold [13]). The temperature, electron density and temporal dependence of the thermophysical properties, C_e, k_e are provided from well-established expressions derived from free electron gas based on the metallic character of the excited electron system [1,17]

$$
\begin{aligned}
&E_F = \frac{(hc)^2}{8m_e c^2}\left(\frac{3}{\pi}\right)^{2/3}(N_e)^{2/3} \\
&F(\varepsilon) = \frac{8\sqrt{2}\pi(m_e)^{2/3}}{h^3}\sqrt{\varepsilon} \\
&\mu(n_e,T_e) = E_F\left[1-\frac{\pi^2}{12}\left(\frac{k_B T_e}{E_F}\right)^2+\frac{\pi^2}{80}\left(\frac{k_B T_e}{E_F}\right)^4\right] \\
&\langle\varepsilon\rangle = \frac{\int_0^\infty \exp\left(-\left((\varepsilon-\mu)/(k_B T_e)+1\right)\right)F(\varepsilon)\varepsilon d\varepsilon}{\int_0^\infty \exp\left(-\left((\varepsilon-\mu)/(k_B T_e)+1\right)\right)F(\varepsilon)d\varepsilon} \\
&C_e(N_e,T_e) = N_e\frac{\partial\langle\varepsilon\rangle}{\partial T_e} \\
&k_e(N_e,T_e) = \frac{1}{3}(u_e)^2\tau_c C_e(N_e,T_e)
\end{aligned}
\qquad (14)
$$

On the other hand, C_L=1.6 J/(cm^{3}K) [1] while the coupling constant is estimated to be $g=g_0(N_e)^{2/3}$, where g_0=0.6×10^{-1}W/(mK) [1].

It is noted that the contribution of ambipolar diffusion of dense electron-hole plasma has not been included in the model. On the one hand, various studies have highlighted the role of ambipolar diffusion in the thermal response of the material and more specifically to studies related to damage threshold evaluation (see for example, studies by Danilov et al [24], and Derrien et al [25] on Silicon). On the other hand, in other studies [13], simulations did not show remarkable changes if the ambipolar diffusion (or transport) were ignored. It is evident though, that the influence might be dependent on the laser parameters (i.e. intensity, pulse duration, fluence and laser wavelength). Therefore this limitation of the present model could be the motivation for the development of a revised version.

Similarly, the temporal length of the pulse is also very important that can set limitations for the use of Eqs.1-14. One major parameter that influences the precision of the calculations is the pulse duration. The electron dynamics and relaxation processes considered in this study assumed an instantaneous thermalisation of the electron system through electron-electron scattering mechanisms. This is an important ingredient towards assuming that the electron system is in thermal equilibrium and T_e is defined. By contrast, for very short pulses (for example, τ_p<100 fs), this argument constitutes an overestimation and therefore alternative techniques to describe electron excitation and relaxation processes are required (see for example Ref. [24] and references therein). Alternative modelling approaches should be used in that case such as quantum mechanics-based approaches to account for the thermalisation of the electron system and

dephasing effects. Similarly, appropriate corrections should be made if pulse durations smaller than the trapping time are used.

D. Fluid dynamics

To model a surface modification following irradiation with mid-IR fs laser pulses, it is assumed that the laser conditions are sufficiently high to result in a phase transition from solid to liquid phase and upon resolidification a periodic relief is induced on the surface of the material based on the series of processes described in the main manuscript. Depending on the laser intensity, mass removal is also possible if the material is heated above a critical temperature (~ $T_L > 1.8 \times T_{boiling} \sim 4000$ for SiO_2 where $T_{boiling} = 2270$ K [21]). The choice of the critical temperature is based on arguments made in previous reports [21,26-28]. More specifically, a solid material subjected to ultrashort pulsed laser heating at sufficiently high fluences undergoes a phase transition to a superheated liquid whose temperature reaches values ~$0.90 \times T_{critical}$ ($T_{critical}$ stands for the thermodynamic critical temperature [27]). In this work, the proposed scenario, of modeling material removal is based on a combination of evaporation of material volumes that exceed upon irradiation lattice temperatures close to $\sim 0.90 \times T_{critical}$ and evaporation due to dynamics of Knudsen layer (adjacent to the liquid-vapor interface [22,25,26]). According to the discussion in Ref. [21], for many materials, a typical value of $T_{critical}$ is 1-2 times higher than the boiling temperature which is $T_{boiling} = 2270$ K for fused silica [21], hence $T_{critical} \sim 2 \times T_{boiling}$ (assuming the smallest value for the attained value of $T_{critical}$). Based on this assumption, the minimum threshold value which should be used is equal to $2 \times 0.9 \times T_{boiling} = 1.8 \times T_{boiling}$. Alternative scenarios for the estimation of the minimum lattice temperature that leads to mass removal could involve a lower temperature, the boiling temperature. It is evident that appropriate experimental setups could assist in a more precise evaluation, however, this is beyond the scope of the present study.

The movement of a material in the molten phase ($T_{melting}$=1988 K [29,30]) is given by the following Navier-Stokes equations (NSE) which describes the dynamics of an uncompressible fluid

$$\rho_0 \left(\frac{\partial \vec{u}}{\partial t} + \vec{u} \cdot \vec{\nabla}\vec{u}\right) = \vec{\nabla} \cdot \left(-P + \mu(\vec{\nabla}\vec{u}) + \mu(\vec{\nabla}\vec{u})^T\right) \quad (15)$$

where ρ_0 and μ stand for the density and viscosity of molten SiO_2, while P and $\vec{u}$ are the pressure and velocity of the fluid. The fluid is considered to be an incompressible fluid (i.e. $\vec{\nabla} \cdot \vec{u} = 0$) .
In regard to the pressure, there are two terms that require special treatment:

- the **recoil pressure** which is related to the lattice temperature of the surface of the material through the equation [31,32]

  $$P_r = 0.54 P_0 \exp\left(L_v \frac{T_L^S - T_{boiling}}{R T_L^S T_{boiling}}\right) \quad (16)$$

  where P_0 is the atmospheric pressure (i.e. equal to 10^5 Pa [33]), L_v is the latent heat of evaporation of the liquid (L_v =7 kJ/gr [33]), R is the universal gas constant, and T_L^S corresponds to the surface temperature. When vapour is ejected, it creates a back (recoil) pressure on the liquid free surface which in turn pushes the melt away in the radial direction[26] which results into a depression of the surface. Furthermore, given the spatially modulated energy deposition on the material, a gradient of the lattice temperature is produced which is, in turn, transferred into the fluid and therefore a capillary fluid convection is produced.

- A precise estimate of the molten material behaviour requires a contribution from the **surface tension related pressure**, P_σ, which is influenced by the surface curvature and is expressed as $P_\sigma = K\sigma$, where K is the free surface curvature and σ=0.310 N/m [34] is the surface tension. The calculation of the pressure associated to the surface tension requires the computation of the temporal evolution of the principal radii of surface curvature R_1 and R_2 that correspond to the

convex and concave contribution, respectively [35]. Hence the total curvature is computed from the expression K=(1/ R_1 +1/ R_2). A positive radius of the melt surface curvature corresponds to the scenario where the centre of the curvature is on the side of the melt relative to the melt surface.

Pressure equilibrium on the material surface implies that the pressure P in Eq.15 should outweigh the accumulative effect of $P_r + P_\sigma$. The thermocapillary boundary conditions imposed at the liquid free surface are the following

$$\frac{\partial u}{\partial Z} = -\sigma/\mu \frac{\partial T_L}{\partial X} \text{ and } \frac{\partial v}{\partial Z} = -\sigma/\mu \frac{\partial T_L}{\partial Y} \quad (17)$$

where (u,v,w) are the components of $\vec{u}$ in Cartesian coordinates. Values for the thermophysical parameters that are used in the simulations are: $\rho_0 = 2.2$ gr/cm^3 [33], μ results from a fitting procedure (Ref.[34,36]) and σ=0.310 N/m [33]. The melting point of fused silica 1988K is taken as the threshold for a phase transition from solid to liquid while the same isothermal is considered as the same criterion for resolidification.

The hydrodynamic equations are solved in regions that contain either solid or molten material. To include the 'hydrodynamic' effect of the solid domain, material in the solid phase is modelled as an extremely viscous liquid (μ_{solid}=10^5 μ), which results into velocity fields that are infinitesimally small. An apparent viscosity is then defined with a smooth switch/step function

$$\delta\left(T_L - T_{melting}\right) = \frac{1}{\sqrt{2\pi}\Delta} e^{-\left[\frac{\left(T_L - T_{melting}\right)^2}{2\Delta^2}\right]} \quad (18)$$

where Δ is in the range of 10-100^{0}K depending on the temperature gradient [21,26].

E. Numerical scheme

To solve the set of the above equations, a scheme based on finite difference method is used. A common approach followed to solve similar problems is the employment of a staggered grid finite difference method which is found to be effective in suppressing numerical oscillations. Unlike the conventional finite difference method, temperatures (T_c and T_L), carrier densities (N_e), pressure (P) are computed at the centre of each element while time derivatives of the displacements and first-order spatial derivative terms are evaluated at locations midway between consecutive grid points. For time-dependent flows, a common technique to solve the Navier-Stokes equations is the projection method and the velocity and pressure fields are calculated on a staggered grid using fully implicit formulations [37,38]. On the other hand, the horizontal and vertical velocities are defined in the centres of the horizontal and vertical cells faces, respectively (for a more detailed analysis of the numerical simulation conditions and the methodology towards the description of fluid dynamics, see Refs. [26,28,30,39-43]).

During the ultrashort period of laser heating, heat loss from the upper surface of target is assumed to be negligible. As a result, a zero heat flux boundary condition is set for the carrier and lattice systems.

- For irradiation with one pulse (*NP=1*), Eqs.1-17 are solved assuming heating of a flat profile; a 2D numerical approach is followed by taking into account the axial symmetry of the problem.

- For *NP>2*, the symmetry breaks and a 2D solution is no longer valid. In that case, a 3D numerical framework is developed (a finite difference methods is used again [26]). The incident beam is no longer perpendicular to the modified profile and therefore the surface geometry influences the spatial distribution of the deposited laser energy. Hence, appropriate modification to the numerical scheme is required to compute energy absorption (for example Eq.7 needs to be corrected). Typical Fresnel equations are used to describe the reflection and transmission of the incident light. Due to multiple reflection and light entrapment, the absorption of the laser beam is modified [44]. The calculation of the pressure associated to the surface tension requires the computation of the

temporal evolution of the principal radii of surface curvature R_1 and R_2 that correspond to the convex and concave contribution, respectively [35]. Hence the total curvature is computed from the expression $K=(1/R_1+1/R_2)$ [26].

In regard to the material removal simulation, in each time step, lattice and carrier temperatures are computed and if lattice temperature reaches $\sim T_L > 1.8 T_{boiling}$, mass removal through evaporation is assumed. In that case, the associated nodes on the mesh are eliminated and new boundary conditions of the aforementioned form on the new surface are enforced. In order to preserve the smoothness of the surface that has been removed and allow an accurate and non-fluctuating value of the computed curvature and surface tension pressure, a fitting methodology is pursued [26].

F. Impact of fluence, pulse duration, laser wavelength on excitation levels.

To evaluate the role of STE states, the electron density and its variation has been calculated as a function of the laser fluence, the pulse duration and the laser wavelength (Figs.S1-S4) for single shot simulations. It is evident that the variation of the electron densities if STE states are formed (Eq.1 in the main manuscript) increases as the higher fluence increases. On the other hand, although simulation results (Figs.S1-S4) indicate that the role of STE can be ignored for small fluences, it is noted that experimental observations have shown that the presence of defects and incubation effects influence significantly the damage threshold of the irradiated material [16,45,46] for multipulse irradiation. Moreover, it is noted that in multiple shot experiments, the change of surface morphology (formation of crests and wells) vary the energy absorption, excited carrier densities and thermal response of the material and therefore a thorough investigation of the impact of repetitive irradiation (including the impact of STE) is required.

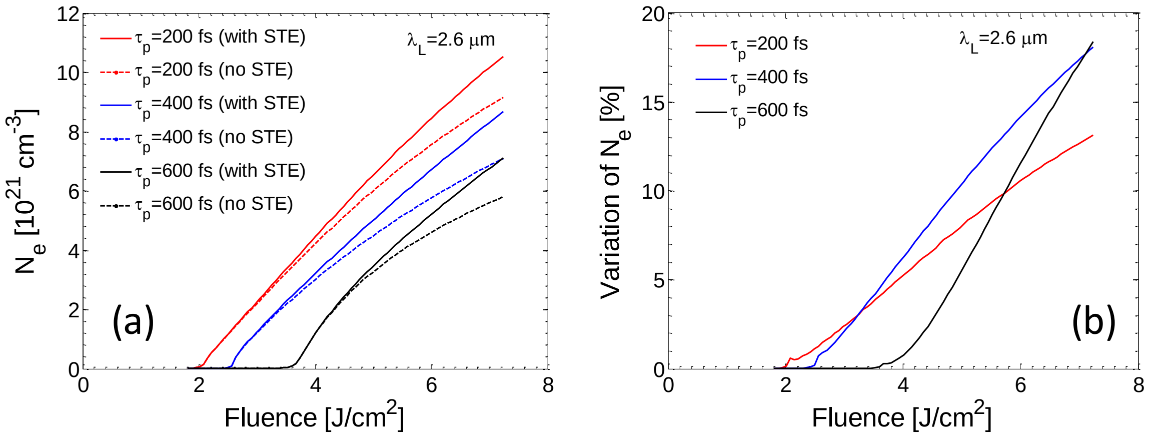

Figure S1. Electron densities (a) and percentage variation (b) with and without STE as a function of fluence for four values of the pulse duration (τ_p=200 fs, 400 fs, 600 fs). Results are shown for λ_L=2.6 μm.

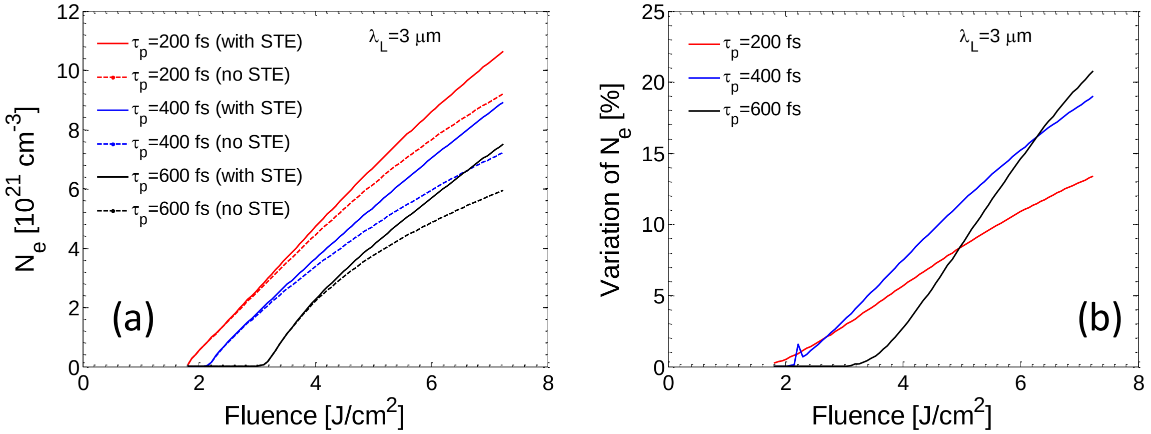

Figure S2. Electron densities (a) and percentage variation (b) with and without STE as a function of fluence for four values of the pulse duration (τ_p=200 fs, 400 fs, 600fs). Results are shown for λ_L=3 μm.

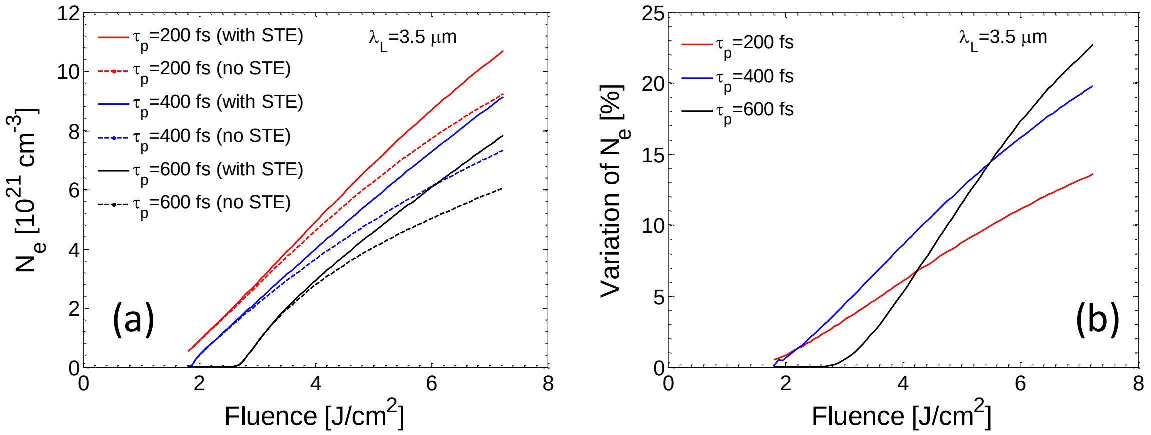

Figure S3. Electron densities (a) and percentage variation (b) with and without STE as a function of fluence for four values of the pulse duration (τ_p=200 fs, 400 fs, 600fs). Results are shown for λ_L=3.5 μm.

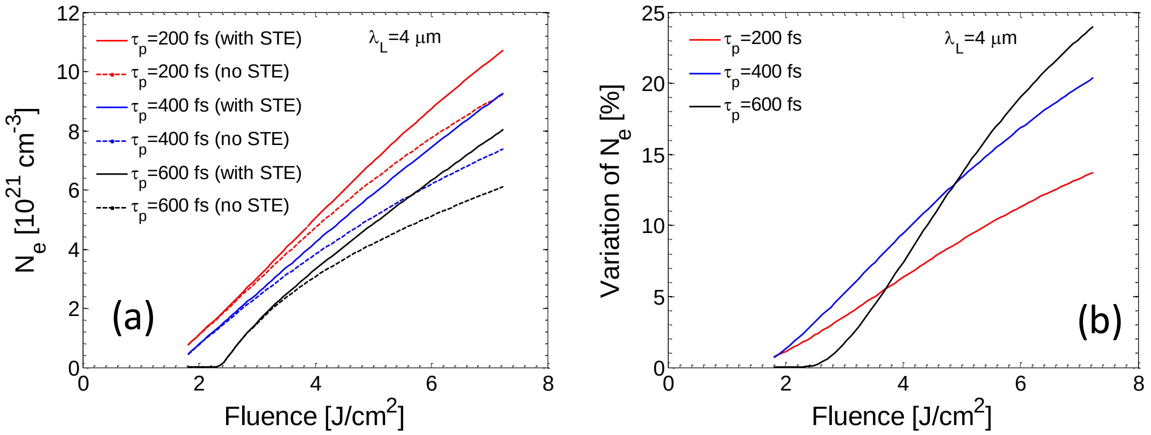

Figure S4. Electron densities (a) and percentage variation (b) with and without STE as a function of fluence for four values of the pulse duration (τ_p=200 fs, 400 fs, 600fs). Results are shown for λ_L=4 μm.

G. SP wavelength *vs. NP*.

Simulations have been performed to compute the variation of the SP wavelength with increasing irradiation dose (*NP*). To compute the SP wavelength, a multiscale approach has been followed to take into account the carrier density for the profile induced as a result of the surface profile after irradiation with the NP^{th}-1 pulse. In Fig.S5, results are shown for λ_L=2.6 μm for SP wavelength as a function of *NP*.

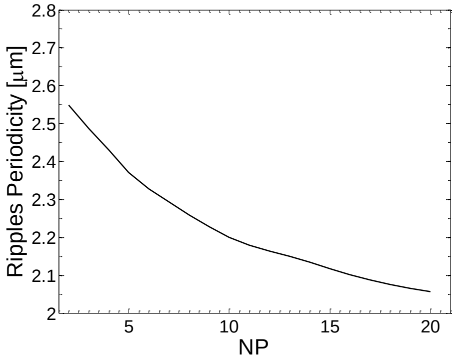

Figure S5. SP wavelength *vs. NP*. Results are shown for λ_L=2.6 μm (laser peak intensity I=1.4×10^{13} W/cm^2).

References

1 Chimier, B. *et al.* Damage and ablation thresholds of fused-silica in femtosecond regime. *Physical Review B* **84**, 094104 (2011).

2 Keldysh, L. V. Ionization in Field of a Strong Electromagnetic Wave. *Sov Phys Jetp-Ussr* **20**, 1307-& (1965).

3 Arnold, D., Cartier, E. & DiMaria, D. J. Acoustic-phonon runaway and impact ionization by hot electrons in silicon dioxide. *Physical Review B* **45**, 1477-1480 (1992).

4 Wu, A. Q., Chowdhury, I. H. & Xu, X. F. Femtosecond laser absorption in fused silica: Numerical and experimental investigation. *Physical Review B* **72**, 085128 (2005).

5 Burakov, I. M. *et al.* Spatial distribution of refractive index variations induced in bulk fused silica by single ultrashort and short laser pulses. *Journal of Applied Physics* **101**, 043506 (2007).

6 Sudrie, L. *et al.* Femtosecond laser-induced damage and filamentary propagation in fused silica. *Physical Review Letters* **89**, 186601 (2002).

7 Tzortzakis, S. *et al.* Femtosecond and picosecond ultraviolet laser filaments in air: experiments and simulations. *Optics Communications* **197**, 131-143 (2001).

8 Sun, Q. *et al.* Measurement of the collision time of dense electronic plasma induced by a femtosecond laser in fused silica. *Optics Letters* **30**, 320-322 (2005).

9 Rudenko, A. *et al.* Spontaneous periodic ordering on the surface and in the bulk of dielectrics irradiated by ultrafast laser: a shared electromagnetic origin. *Sci Rep-Uk* **7**, 12306 (2017).

10 Penano, J. R., Sprangle, P., Hafizi, B., Manheimer, W. & Zigler, A. Transmission of intense femtosecond laser pulses into dielectrics. *Physical Review E* **72**, 036412(2005).

11 Mirza, I. *et al.* Ultrashort pulse laser ablation of dielectrics: Thresholds, mechanisms, role of breakdown. *Sci Rep-Uk* **6**, 39133 (2016).

12 Mézel, C. *et al.* Formation of nanocavities in dielectrics: A self-consistent modeling. *Physics of Plasmas* **15**, 093504 (2008).

13 Rämer, A., Osmani, O. & Rethfeld, B. Laser damage in silicon: Energy absorption, relaxation, and transport. *Journal of Applied Physics* **116**, 053508 (2014).

14 Kudryashov, S. *Dynamic interplay between femtosecond laser ionization mechanisms in solid dielectrics*. Vol. 5991 LD (SPIE, 2006).

15 Kudryashov, S. *Unified model of femtosecond laser ionization in bulk solid dielectrics*. Vol. 6108 PWL (SPIE, 2006).

16 Mao, S. S. *et al.* Dynamics of femtosecond laser interactions with dielectrics. *Applied Physics a-Materials Science & Processing* **79**, 1695-1709 (2004).

17 Tsibidis, G. D., Skoulas, E., Papadopoulos, A. & Stratakis, E. Convection roll-driven generation of supra-wavelength periodic surface structures on dielectrics upon irradiation with femtosecond pulsed lasers. *Physical Review B* **94**, 081305(R) (2016).

18 Malitson, I. H. Interspecimen Comparison of Refractive Index of Fused Silica. *Journal of the Optical Society of America* **55**, 1205-& (1965).

19 Dufft, D., Rosenfeld, A., Das, S. K., Grunwald, R. & Bonse, J. Femtosecond laser-induced periodic surface structures revisited: A comparative study on ZnO. *Journal of Applied Physics* **105**, 034908 (2009).

20 Anisimov, S. I., Kapeliovich, B. L. & Perel'man, T. L. Electron-emission from surface of metals induced by ultrashort laser pulses. *Zhurnal Eksperimentalnoi Teor. Fiz.* **66**, 776-781 (1974 [Sov. Phys. Tech. Phys. 11, 945 (1967)]).

21 Burakov, I. M., Bulgakova, N. M., Stoian, R., Rosenfeld, A. & Hertel, I. V. Theoretical investigations of material modification using temporally shaped femtosecond laser pulses. *Applied Physics a-Materials Science & Processing* **81**, 1639-1645 (2005).

22 Wortmann, D., Ramme, M. & Gottmann, J. Refractive index modification using fs-laser double pulses. *Optics Express* **15**, 10149-10153 (2007).

23 Richter, S. *et al.* Nanogratings in fused silica: Formation, control, and applications. *Journal of Laser Applications* **24**, 042008 (2012).

24 Danilov, P. *et al.* Electron-ion coupling and ambipolar diffusion in dense electron-hole plasma in thin amorphous Si films studied by single-shot, pulse-width dependent ultrafast laser ablation. *Applied Surface Science* **425**, 170-175 (2017).

25 Derrien, T. J.-Y. & Bulgakova, N. *Modeling of silicon in femtosecond laser-induced modification regimes: accounting for ambipolar diffusion*. Vol. 10228 EOO (SPIE, 2017).

26 Tsibidis, G. D., Barberoglou, M., Loukakos, P. A., Stratakis, E. & Fotakis, C. Dynamics of ripple formation on silicon surfaces by ultrashort laser pulses in subablation conditions. *Physical Review B* **86**, 115316 (2012).

27 Kelly, R. & Miotello, A. Comments on explosive mechanisms of laser sputtering. *Applied Surface Science* **96-98**, 205-215 (1996).

28 Tsibidis, G. D. *et al.* Modelling periodic structure formation on 100Cr6 steel after irradiation with femtosecond-pulsed laser beams. *Applied Physics A* **124**, 27 (2017).

29 Jiang, L. & Tsai, H. L. A plasma model combined with an improved two-temperature equation for ultrafast laser ablation of dielectrics. *Journal of Applied Physics* **104**, 093101 (2008).

30 Papadopoulos, A., Skoulas, E., Tsibidis, G. D. & Stratakis, E. Formation of periodic surface structures on dielectrics after irradiation with laser beams of spatially variant polarisation: a comparative study. *Applied Physics A* **124**, 146 (2018).

31 Zhao, H. Y. *et al.* Modelling of keyhole dynamics and porosity formation considering the adaptive keyhole shape and three-phase coupling during deep-penetration laser welding. *Journal of Physics D-Applied Physics* **44**, 485302 (2011).

32 Cho, J. H., Farson, D. F., Milewski, J. O. & Hollis, K. J. Weld pool flows during initial stages of keyhole formation in laser welding. *Journal of Physics D-Applied Physics* **42**, 175502 (2009).

33 Doualle, T. *et al.* Thermo-mechanical simulations of CO2 laser-fused silica interactions. *Journal of Applied Physics* **119**, 113106 (2016).

34 Boyd, K., Ebendorff-Heidepriem, H., Monro, T. M. & Munch, J. Surface tension and viscosity measurement of optical glasses using a scanning CO2 laser. *Optical Materials Express* **2**, 1101-1110 (2012).

35 Spivak, M. *A comprehensive introduction to differential geometry*. 3rd edn, (Publish or Perish, Inc., 1999).

36 Urbain, G., Bottinga, Y. & Richet, P. Viscosity of Liquid Silica, Silicates and Alumino-Silicates. *Geochim Cosmochim Ac* **46**, 1061-1072 (1982).
37 Fletcher, C. A. J. & Srinivas, K. *Computational techniques for fluid dynamics*. 2nd edn, (Springer-Verlag, 1991).
38 Wang, Y. & Tsai, H. L. Impingement of filler droplets and weld pool dynamics during gas metal are welding process. *International Journal of Heat and Mass Transfer* **44**, 2067-2080 (2001).
39 Margiolakis, A., Tsibidis, G. D., Dani, K. M. & Tsironis, G. P. Ultrafast dynamics and sub-wavelength periodic structure formation following irradiation of GaAs with femtosecond laser pulses. *Physical Review B* **98**, 224103 (2018).
40 Tsibidis, G. D., Fotakis, C. & Stratakis, E. From ripples to spikes: A hydrodynamical mechanism to interpret femtosecond laser-induced self-assembled structures. *Physical Review B* **92**, 041405(R) (2015).
41 Tsibidis, G. D., Skoulas, E. & Stratakis, E. Ripple formation on nickel irradiated with radially polarized femtosecond beams. *Optics Letters* **40**, 5172-5175 (2015).
42 Tsibidis, G. D. & Stratakis, E. Ripple formation on silver after irradiation with radially polarised ultrashort-pulsed lasers. *Journal of Applied Physics* **121**, 163106 (2017).
43 Zuhlke, C. A. *et al*. Investigation of femtosecond laser induced ripple formation on copper for varying incident angle. *AIP Advances* **8**, 015212 (2018).
44 Solana, P. & Negro, G. A study of the effect of multiple reflections on the shape of the keyhole in the laser processing of materials. *Journal of Physics D-Applied Physics* **30**, 3216-3222 (1997).
45 Rosenfeld, A., Lorenz, M., Stoian, R. & Ashkenasi, D. Ultrashort-laser-pulse damage threshold of transparent materials and the role of incubation. *Applied Physics a-Materials Science & Processing* **69**, S373-S376 (1999).
46 Ashkenasi, D., Lorenz, M., Stoian, R. & Rosenfeld, A. Surface damage threshold and structuring of dielectrics using femtosecond laser pulses: the role of incubation. *Applied Surface Science* **150**, 101-106 (1999).
